# Supplementary material for: Laboratory Evolution Reveals Transcriptional Mechanisms Underlying Thermal Adaptation of Escherichia coli
Source: Genome Biol Evol. 2025 Oct 3;17(10):evaf171. doi: 10.1093/gbe/evaf171 (PMC12492005; doi:10.1093/gbe/evaf171)
Supplement: evaf171_Supplementary_Data [file evaf171_supplementary_data.zip › Hot TALE Manuscript - GBE R0 - SI.pdf]

Supplemental Figures

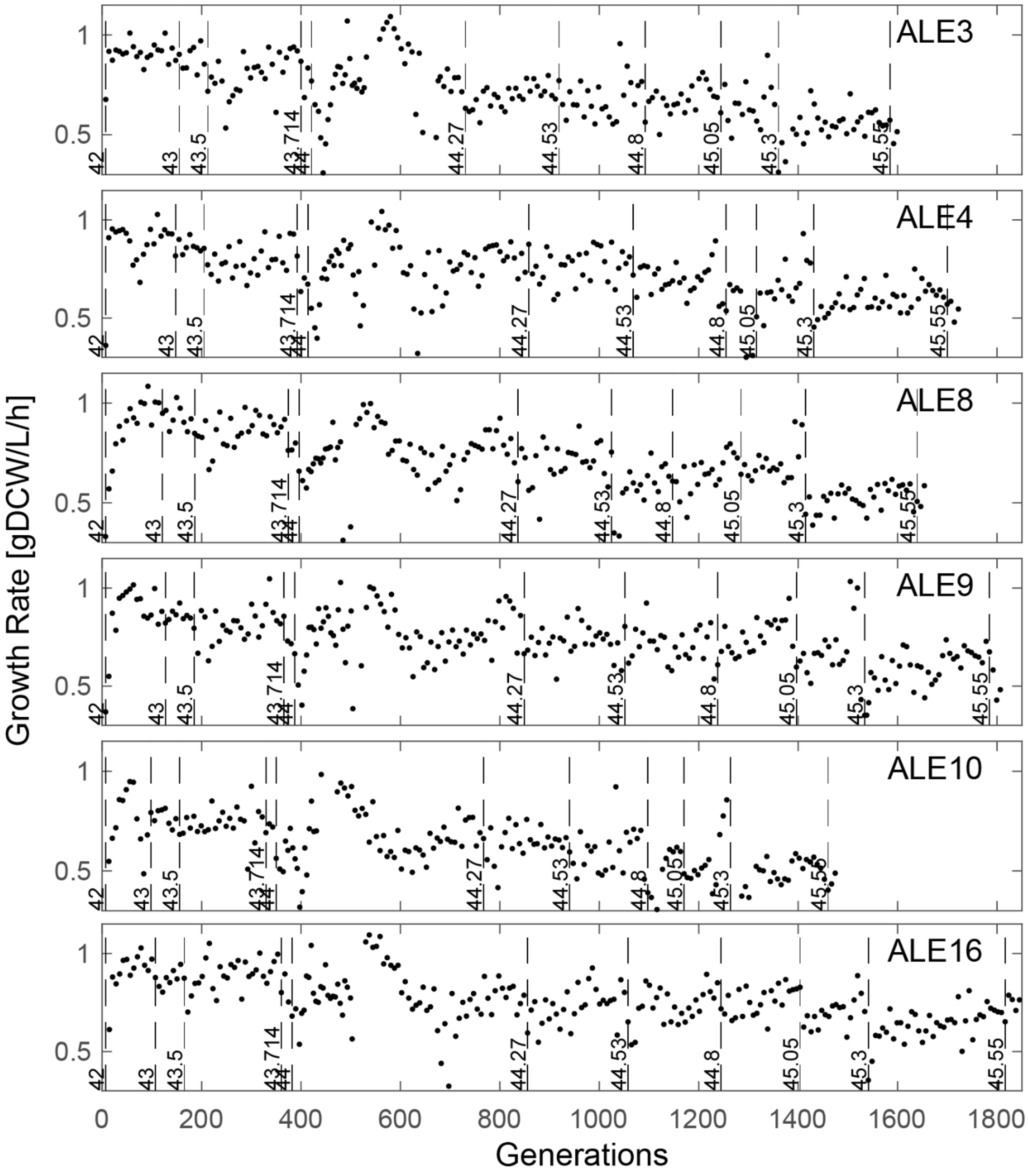

Figure S1. Growth rates and temperatures for each flask in ALE. Dotted vertical lines indicate the flasks at which the temperature was increased. Generation numbers are estimated from the growth rate and elapsed time of each flask.

Supplemental Tables

Table S1: Mutations in 42c_3 starting strain.

Mutations details, position, type, sequence change, and affected genes were generated by the ALEdb mutation calling pipeline (Phaneuf et al. 2019). Positions refer to locations in the NC_000913 genome (Hayashi et al. 2006). The 'Figure' column contains the figure number in which a mutation is discussed, with a * for *mutL* which is discussed for its relevance to the hypermutator phenotype but not in a particular figure. This is a subset of **Table S2** that only includes the mutations that were present in the starting strain.

Table S2. Mutations in high temperature tolerized strains.

Mutations details, position, type, sequence change, and affected genes were generated by the ALEdb mutation calling pipeline (Phaneuf et al. 2019). 'Category' was used to generate **Figure 1C.** The 'Figure' column is blank for mutations that are not discussed in the paper, and references a figure number otherwise (* for *mutL*, which is discussed but not in a figure). Columns labeled with strain numbers indicate presence or absence of the mutation in the given strain.

Table S3. iModulon activities in high temperature tolerized strains.

iModulons are listed by explained variance in the evolved strains' data. If the iModulon was significantly differentially activated in **Figure 1F**, 'Evolution P' indicates the false discovery rate corrected p-value and 'Evolved Minus WT' shows the difference in mean activities. Similarly, if the iModulon was significantly differentially activated in **Figure 1G**, then statistics are listed under 'Temperature P' and '44C Minus 30C' where positive differences indicate upregulation at 44°C relative 30°C. 'Category' was used to generate **Figure 1D**. 'Figure' lists the figure that is relevant for understanding the iModulon's behavior, if applicable. Remaining descriptive columns are copied from the PRECISE-1K curation of these iModulons (Lamoureux et al. 2023). See iModulonDB.org for details of each iModulon, including its member genes, activity levels across over 1000 conditions including those from this study, and overlap with associated regulons.

Table S4. Meaning, evidence, and novelty for each proposed connection

Each row of this table corresponds to an arrow in **Figure 2**, as well as **3D, 4C, 5K-L,** and **6B**. The 'From', 'To', and 'Meaning' columns describe the relationship in more detail than can be displayed on a knowledge graph. 'Figure' contains the specific relevant figure panels if available. 'Data Evidence' contains written descriptions of the evidence from this study associated with the relationship. 'Literature Evidence' lists citations which agree with or establish the relationship. The 'New?' column assesses the novelty of the relationship, which may be previously established, new in this study, or describe the new details and perspectives revealed by comparing the literature with the new data.
